# Adaptation of the autotrophic acetogen *Sporomusa ovata* to methanol accelerates the conversion of CO_2_ to organic products

**DOI:** 10.1038/srep16168

**Published:** 2015-11-04

**Authors:** Pier-Luc Tremblay, Daniel Höglund, Anna Koza, Ida Bonde, Tian Zhang

**Affiliations:** 1 The Novo Nordisk Foundation Center for Biosustainability, Technical University of Denmark, Hørsholm, Denmark

## Abstract

Acetogens are efficient microbial catalysts for bioprocesses converting C1 compounds into organic products. Here, an adaptive laboratory evolution approach was implemented to adapt *Sporomusa ovata* for faster autotrophic metabolism and CO_2_ conversion to organic chemicals. *S. ovata* was first adapted to grow quicker autotrophically with methanol, a toxic C1 compound, as the sole substrate. Better growth on different concentrations of methanol and with H_2_-CO_2_ indicated the adapted strain had a more efficient autotrophic metabolism and a higher tolerance to solvent. The growth rate on methanol was increased 5-fold. Furthermore, acetate production rate from CO_2_ with an electrode serving as the electron donor was increased 6.5-fold confirming that the acceleration of the autotrophic metabolism of the adapted strain is independent of the electron donor provided. Whole-genome sequencing, transcriptomic, and biochemical studies revealed that the molecular mechanisms responsible for the novel characteristics of the adapted strain were associated with the methanol oxidation pathway and the Wood-Ljungdahl pathway of acetogens along with biosynthetic pathways, cell wall components, and protein chaperones. The results demonstrate that an efficient strategy to increase rates of CO_2_ conversion in bioprocesses like microbial electrosynthesis is to evolve the microbial catalyst by adaptive laboratory evolution to optimize its autotrophic metabolism.

Acetogenic bacteria have a unique autotrophic metabolism that is being exploited for the development of biotechnologies aiming at the production of commodity chemicals from C1 compounds like CO_2_ and CO^1^,^2^. During growth on C1 compounds, CO_2_ is used as an electron acceptor and is being reduced to acetyl-CoA via the Wood-Ljungdahl pathway^3^,^4^. Acetogens have been mainly employed as microbial catalyst for the production of biofuels from the fermentation of syngas generated from industrial or municipal wastes^5^. They also have been used for the production of commodity chemicals from CO_2_ and electricity by microbial electrosynthesis (MES) in a bioelectrochemical reactor^6^,^7^,^8^.

Recently, the multiplication of genome sequences and the development of genetic systems for engineering several acetogens led to important progress in the characterization of their metabolism and in the construction of microbial catalysts for industrial processes^1^,^9^,^10^,^11^,^12^,^13^,^14^. Adaptive laboratory evolution (ALE) which is initiated by applying an environmental stress on a microbial population to select for fitter mutants^15^ can also be a powerful approach to develop readily beneficial phenotypic characteristics in industrial strains^16^,^17^. For example, ALE has enabled the development of bacterial strains more tolerant to products of interest like biofuels^18^,^19^,^20^ or faster at reducing electron acceptors involved in bioremediation processes^21^. Furthermore, ALE in combination with whole-genome sequencing and transcriptomic sutdies is an efficient strategy to gain useful insights on bacterial metabolism^17^,^22^.

Methanol is a promising C1 compounds used as substrate for several bioprocesses producing organic molecules (Schrader, 2009). Methanol can be oxidized anaerobically by acetogens with CO_2_ from the environment as electron acceptor^4^,^23^,^24^. Metabolic breakdown by bacteria is a well-known route for the quick elimination of toxic compounds with the purpose of limiting exposure^25^. Therefore, methanol, which is a solvent inhibiting bacterial growth^26^,^27^,^28^, is a perfect substrate for an ALE experiment aiming at applying a pressure to select acetogenic strains with faster autotrophic metabolism and higher tolerance to solvent.

In this study, ALE was employed to develop a strain of the acetogen *Sporomusa ovata* growing significantly faster on methanol. The autotrophic metabolism of the adapted strain was also investigated with H_2_ as the electron donor. Since *S. ovata* is one of the most efficient electroautotrophic species reported in the literature^29^,^30^,^31^, the methanol-adapted strain was tested in a MES system to verify if a faster autotrophic metabolism translated in better performance for the electroconversion of CO_2_ into acetate. Whole-genome sequencing, transcriptomic, and biochemical studies were used to uncover the molecular mechanisms responsible for the improvement of the autotrophic metabolism and for the higher tolerance to methanol.

## Results and Discussion

### Adaptation of *S. ovata* for faster growth on methanol

**Figure 1:**
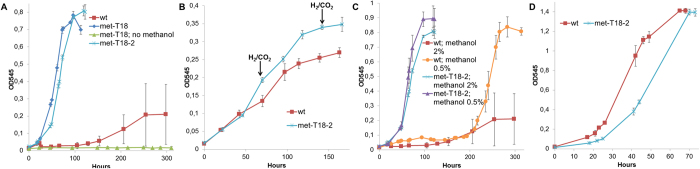
Autotrophic growth and heterotrophic growth of the methanol-adapted *S. ovata*. (**A**) Growth on 2% methanol with *S. ovata* wild type (wt), a representative methanol-adapted culture after 18 transfers (met-T18) in the presence or in the absence of methanol, and a clone isolated from the adapted culture #2 (met-T18-2). (**B**) Growth on H_2_-CO_2_. The black arrows indicate repressurization at 1.7 atm of the H_2_-CO_2_ atmosphere. (**C**) Growth on different concentration of methanol. (**D**) Growth with 40 mM betaine. Each curve is the mean of three replicates.

Strains of *S. ovata* capable of growing faster with methanol as the sole source of carbon and electrons were developed by ALE (Fig. 1A). *S. ovata* was chosen for this study because its genome has recently been sequenced and also because it is an efficient microbial catalyst for biotechnologies like MES using C1 compounds as substrate^12^,^29^,^30^. The wild type *S. ovata* was initially cultivated for two independent adaptation experiments in defined minimal medium with 2% methanol. After a lag phase of 105.7 ± 7.2 hours, growth began and a 10% inoculum was transferred into fresh methanol medium. Subsequent transfers were done after the cultures reached the mid-log phase to select for fitter mutants. The ALE experiment was completed over a period of two months and stopped after 18 transfers since no further improvement of the growth rate was observed. A resulting representative adapted culture was growing 5 times faster than the wild type with a doubling time of 13.0 ± 1.1 hours compared to 64.6 ± 19.4 hours. Clones isolated from the adapted cultures had comparable growth rates. A representative clone from the adapted culture #2 (met-T18-2) (Fig. 1A) with a doubling time of 13.2 ± 0.4 hours was further characterized.

### Growth of methanol-adapted *S. ovata* on H_2_-CO_2_, 0.5% methanol, and betaine

**Figure 2:**
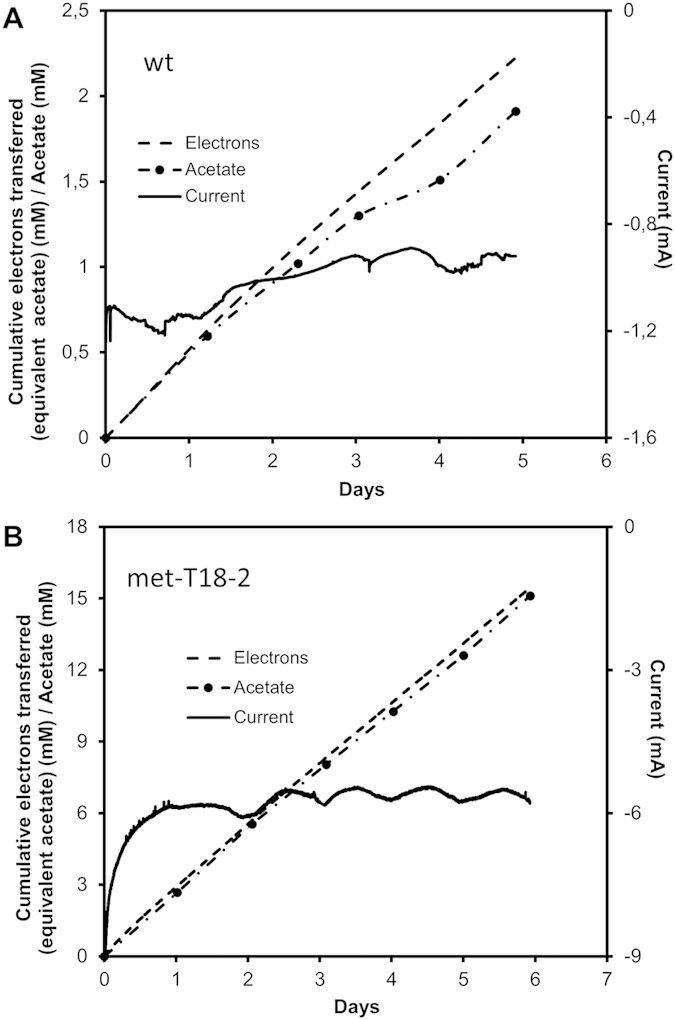
Microbial electrosynthesis of acetate from CO_2_. Electron transferred, acetate and current production with (**A**) *S. ovata* wild type and (**B**) the methanol-adapted *S. ovata* met-T18-2. Electron transferred curves correspond to the acetate concentration in mM if all the electrons transferred were converted to acetate. Acetate production curves in mM correspond to the real progression of acetate concentration in the MES reactor detected by high-pressure liquid chromatography. Results shown are from a representative example of three replicate MES reactors.

The growth of acetogens on methanol requires the reduction of CO_2_ through the Wood-Ljundahl pathway^4^,^32^. To determine if met-T18-2 could also grow better autotrophically with another electron donor than methanol, it was cultivated in a minimal medium with H_2_ and CO_2_ only. At the end of the log phase, the OD545 of the methanol-adapted strain was 0.32 ± 0.00 compared to 0.24 ± 0.01 for the wild type indicating a higher capacity for biomass generation from CO_2_ (Fig. 2B). This observation suggests that the adapted strain became more efficient at reducing CO_2_ through its central metabolism or more efficient at synthetizing cellular components in autotrophic growth conditions.

To establish if the adapted strain develops a greater tolerance to methanol toxicity, the wild type and the adapted strain were grown with methanol at a lower concentration of 0.5%. As expected, a higher concentration of methanol was more toxic for *S. ovata* since the wild type, despite a long lag phase, was growing 3.2-fold faster on 0.5% methanol with a doubling time of 20.9 ± 0.5 hours and 4-fold increase in maximum OD545 compared to 2% methanol (Fig. 2C). In the case of met-T18-2, a slightly better doubling time of 12.2 ± 0.2 hours and a comparable final OD545 were observed with 0.5% methanol versus 2% methanol. These results indicate that the adapted strain is fitter than the wild type at both high and low methanol concentrations. More importantly, the adapted strain clearly developed mechanisms to be more tolerant to methanol toxicity since it grows similarly at high and low methanol concentrations contrary to the wild type.

To verify if the faster growth phenotype associated to the methanol-adapted strain is specific to autotrophic growth and not to a faster metabolism in general, the wild type and the adapted strain were grown on betaine which is the carbon and electron source generally employed for heterotrophic growth and maintenance of *S. ovata* cultures in the laboratory (Fig. 2D). With betaine, the adapted strain had a doubling time of 12.4 ± 0.3 hours and was growing slower than the wild type which had a doubling time of 8.4 ± 0.1 hours. These observations suggest that the adaptation of *S. ovata* metabolism for autotrophic growth on methanol has been done at the expense of its growth efficiency on heterotrophic substrate like betaine.

### MES of acetate from CO_2_

One of the most promising features of *S. ovata* as a microbial catalyst for bioprocesses is its capacity to reduce CO_2_ to organic compounds with electrons coming from the cathode of a MES reactor^6^,^31^. Interestingly, met-T18-2 was 6.5 times more efficient at reducing CO_2_ in a MES system equipped with a graphite cathode poised at a potential of −690 mV versus SHE than the wild type with an average acetate production rate of 866.7 ± 373.5 mM m^−2^ day^−1^ compared to 133.5 ± 43.2 mM m^−2^ day^−1^ (Fig. 2). As expected, the average current consumption density of −2161.7 ± 1071.4 mA m^−2^ observed with the adapted strain was 6.8-fold higher than with the wild type −320.1 ± 98.5 mA m^−2^. Electron recovery in acetate was also slightly higher in the adapted strain 94.7 ± 2.1% compared to the wild type 85.3 ± 8.3%. These observations provide additional evidence that the methanol-adapted strain has become more efficient at reducing CO_2_ under diverse autotrophic conditions including the ones prevailing during MES. Furthermore, the results presented here clearly demonstrate that the autotrophic metabolism of microbial catalysts was an important bottleneck for previously published MES systems^30^ and that optimization via ALE or other molecular biology approaches can improve significantly MES performance.

### The involved molecular mechanisms

**Figure 3:**
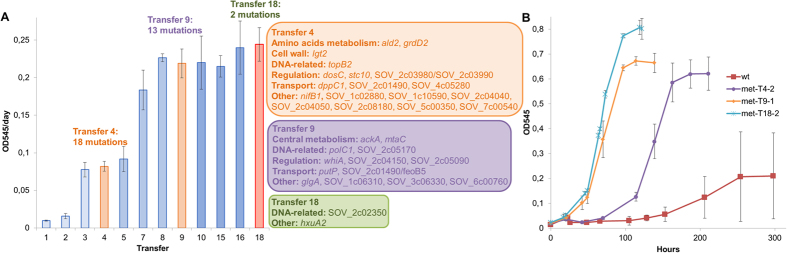
The ALE experiment. (**A**) Growth rate at different transfers on 2% methanol and mutations detected after jump in the growth rate at transfer 4 and 9 and at the final transfer 18. (**B**) Growth curves on 2% methanol with clones isolated from transfer 4, 9, 18, and the wild type. Each value and curve is the mean of three replicates.

Whole genome sequencing was used to identify the mutations associated with the observed improvement in the autotrophic metabolism as well as with the higher tolerance to methanol. Isolated clones from two independent *S. ovata* adapted cultures after the 18^th^ transfer on 2% methanol were sequenced and had 33 mutations in common (Fig. 3A, Table S1). To establish a clear correlation between those mutations and the observed improvement in the growth rate, genomes of clones isolated from the 4^th^ transfer and the 9^th^ transfer were also sequenced. These transfers were selected because they followed transfers where significant jump in growth rate were observed (Fig. 3). A clone isolated from the 4^th^ transfer had a longer lag phase and a doubling time of 23.5 ± 1.0 hours which is 2.7 times better than the wild type, but still significantly slower than transfer 18 (Fig. 3B). A clone isolated from the 9^th^ transfer had a lag phase and a doubling time of 16.3 ± 0.5 hours closer to the doubling time observed at transfer 18. Among the 33 mutations found in the 2 independent adapted cultures sequenced from the transfer 18, 18 mutations were involved in the first jump observed before transfer 4 and 13 mutations appeared in the second jump before transfer 9. The relative large number of mutations found at transfer 4 combines with the mutations detected at transfer 9 and the two mutations appearing later suggest that a complex network of synergistic effects is required to improve the metabolism of *S. ovata* sufficiently to simultaneously increase its tolerance to methanol and its capacity for autotrophic growth.

Some of the 33 common mutations identified may not be specifically related to faster growth on 2% methanol. Repeated exposure to exponential phase may have resulted in the application of a selective pressure for mutations increasing cell fitness during this growth phase. Nevertheless, their presence in two independent experiments suggests that even if those mutations could have appeared with other electron donors following serial transfer during the exponential growth phase, they are still important for faster growth and metabolism of *S. ovata* including for methanol-dependent growth as well as other autotrophic growth conditions. Because of the absence of a genetic system in *S. ovata*, it is impossible to establish clearly causality between the detected mutations and the observed phenotypes. Thus, based on biochemical evidences as well as the current state of knowledge several common mutations possibly of higher importance will be further discussed in regards to their involvement in faster autotrophic growth and higher tolerance to methanol.

Among the 33 common mutations identified, some were located in genes involved in DNA replication and rearrangement. At transfer 4, the gene *topB2* coding for one of the two topoisomerase III of *S. ovata* contained a 1bp deletion (Table S1). In *E. coli*, *topB* mutants present higher frequencies of frameshift mutations^33^,^34^. At transfer 9, the gene *polC1* coding for one of the two DNA polymerase III of *S. ovata* was mutated. A single nucleotide polymorphism (SNP) caused the substitution of a glutamic acid by a lysine at position 587 in the polymerase/histidinol phosphatase-like domain (PHP) of the enzyme. In the DNA polymerase III of *Thermus thermophilus* and *Thermus aquaticus*, this domain has an exonuclease activity and is thought to proofread freshly-synthesized DNA^35^,^36^. The PHP domain of *S. ovata* PolC1 has all the metal-coordinating residues required to carry out exonuclease activity and could also be involved in proofreading^37^. The mutations in *topB2* and *polC1* are possible causes of increase in mutation frequencies accelerating the adaptation of *S. ovata* to methanol.

### Differential expression in the methanol-adapted *S. ovata*

Mutations were found in front or into genes coding for proteins participating to the regulatory network of *S. ovata* including 4 transcriptional factors (Stc10, SOV_2c03990, WhiA, and SOV_2c04150) and 2 proteins (DosC and SOV_2c05090) involved in the synthesis of cyclic di-GMP^38^ (Fig. 3; Table S1). The combination of those mutations might have a significant effect on the transcriptome of the methanol-adapted strains. For instance, the complete disruption of the GGDEF domain of DosC due to a 1 bp deletion and the SNP located in the GGDEF domain of SOV_2c05090 could impact on the intracellular level of cyclic di-GMP, a second messenger involved in the transcriptional regulation of many cellular processes including biofilm formation and motility^39^. Based on these observations, RNA sequencing was used to compare gene expression on H_2_-CO_2_ in the methanol-adapted strain met-T18-2 to the wild type (Table S2 and S3). Genes that were found to be differentially-expressed were involved in central metabolism and in biosynthetic pathways associated with autotrophic growth in minimal medium. Other differentially-expressed genes were implicated in processes potentially related to an increase in the tolerance to methanol such as cell wall maintenance, membrane transport, amino acids biosynthesis, and protein folding.

### Adaptation of the central metabolism

**Figure 4:**
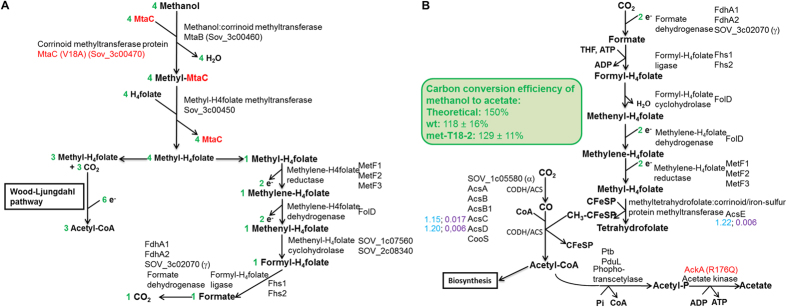
Modifications in the central metabolism of the methanol-adapted *S. ovata* strain. (**A**) The methanol oxidation pathway and (**B**) the Wood-Ljungdahl pathway of *S. ovata*. Mutated genes are in red. Log_2_ fold in blue and q-value in purple are indicated beside differentially-expressed genes in the adapted strain compared to the wild type grown with H_2_-CO_2_. A log_2_ fold change ≥ 1.15 or ≤ –1.15 and a q-value ≤ 0.02 were used as cutoff. Carbon conversion efficiency was established experimentally from three culture replicates on 0.5% methanol.

The pathway for methanol oxidation in acetogens has been partially characterized in the thermophile *Moorella thermoacetica*^4^,^40^. In *S. ovata*, biochemical evidences demonstrated that the first step of methanol oxidation is catalyzed by a corrinoid-dependent methyltransferase that transfers the methyl group of methanol to tetrahydrofolate (H_4_folate)^24^,^41^. A recent proteomic study identified the two components of the corrinoid-dependent methyltransferase in the closely-related species *Sporomusa* strain An4^42^. It consists of MtaC, a corrinoid methyltransferase protein, and MtaB, a methanol:corrinoid methyltransferase that transfers the methyl group of methanol to the corrinoid bound to MtaC. Visser *et al.*, 2015 also suggested that the second step of methanol oxidation was catalyzed by a methyl-H_4_folate-methyltransferase that transfers the methyl group from methyl-MtaC to H_4_folate. Subsequently, methyl group from methanol oxidation entered the Wood-Ljungdahl pathway in combination with CO_2_ serving as electron acceptor to form acetyl-CoA^4^. *S. ovata* has genes coding for enzymes catalyzing all the steps of the methanol oxidation pathway and of the Wood-Ljungdahl pathway^12^ (Fig. 4).

The predicted carbon conversion efficiency to acetate of the anaerobic methanol oxidation pathway is 150% with 2 moles of reduced CO_2_ coming from the environment for every 4 moles of methanol consumed. With 0.5% methanol as the substrate, carbon conversion efficiencies were 129 ± 11% for the adapted strain and 118 ± 16% for the wild type. The difference with the predicted carbon conversion efficiency can be explained by the assimilation of a fraction of the carbon coming from methanol and CO_2_ into the biomass. As expected, the adapted strain produced acetate faster than the wild type. The acetate production rate was 6.1 ± 0.4 mM day^−1^ for the adapted strain with a methanol consumption of 9.5 ± 1.1 mM day^−1^ compared to the wild type producing 2.6 ± 0.2 mM day^−1^ of acetate and consuming 4.4 ± 0.8 mM day^−1^ of methanol.

Mutations and differential expression of genes of the methanol oxidation pathway and of the Wood-Ljungdahl pathway that could contribute to the development of more efficient autotrophic metabolism were found in the adapted strains. A SNP causing the substitution of a valine by an alanine in the N-terminal corrinoid-binding domain of MtaC was detected at transfer 9 (Fig. 4A, Table S1). Like in *Sporomusa* stain An4, MtaC of *S. ovata* is probably involved in the first step of methanol oxidation and its mutation suggests that this protein has been optimized for more efficient methanol oxidation and faster growth.

The gene *ackA* coding for the acetate kinase responsible for the conversion of acetyl-phosphate to acetate in the Wood-Ljungdahl pathway^3^,^43^ was also mutated at transfer 9. A SNP caused the substitution of the arginine at position 176 to a glutamine (Table S1, Fig. 4B). To establish if this mutation had an impact on the acetate kinase, the acetate-forming activity of the enzyme was measured^44^ in whole cell lysates of the wild type and of the adapted strain (met-T18-2) grown on H_2_-CO_2_. The acetate kinase activity was 1.5 times higher in the adapted strain with 204.5 ± 29.4 nmol of NADP^+^ reduced min^−1^ mg protein^−1^ compared to 137.5 ± 19.3 for the wild type. No statistically-significant differential expression was detected for *ackA* between the adapted strain and the wild type. This suggests that the mutation in AckA has a positive effect on the enzyme performance or stability that could lead to improvement of the efficiency of the Wood-Ljungdahl pathway.

Higher transcript abundance was detected for genes of the Wood-Ljungdahl pathway coding for the methyltetrahydrofolate:corrinoid/iron-sulfur protein methyltransferase AcsE, for the corrinoid/iron-sulfur protein large subunit AcsC, and for the corrinoid/iron-sulfur protein small subunit AcsD (Fig. 4B, Table S2). AcsE catalyzes the transfer of the methyl group acquired by the tetrahydrofolate cofactor from methanol to the cobalamin cofactor of the corrinoid iron sulfur protein (CFeSP) encoded by *acsC* and *acsD*^43^. This reaction is at the heart of the Wood-Ljungdahl pathway and higher expression of *acsCDE* might translate into better autotrophic metabolism in the methanol-adapted *S. ovata* strain.

*acsCDE* are predicted to be encoded within the same operon with other differentially expressed genes including *hdrC* which is coding for the C subunit of the heterodisulfide reductase. *hdrA*, the gene coding for the A subunit of the heterodisulfide reductase which is located elsewhere in the genome (Table S2) had also higher transcript abundance in met-T18-2. In methanogens, the heterodisulfide reductase is involved in a flavin-based electron bifurcation mechanism coupling the reduction of CoM-S-S-CoM to the reduction of ferredoxin with electrons from H_2_^45^. Although its exact function is not known, the transcriptomic data presented here suggests that the heterodisulfide reductase of *S. ovata* could also be implicated in autotrophic metabolism.

### Pyrimidines and vitamins biosynthesis

Many genes coding for enzymes participating in biosynthetic pathways were differentially expressed in met-T18-2. This included the eight most highly-upregulated genes all found in the same operon involved in pyrimidine biosynthesis (Table S2). In the laboratory, *S. ovata* is maintained heterotrophically in a medium amended with 0.1% yeast extract which is known to contain substantial amount of pyrimidine^46^. During the ALE experiment, the minimal medium used for methanol adaptation did not contain yeast extract and thus *S. ovata* cells had to increase their capacity for *de novo* pyrimidine biosynthesis. Pyrimidine nucleotides are involved in many cellular processes including the biosynthesis of DNA, RNA, coenzymes, and of components of the cell wall^47^.

Genes related to the biosynthesis of thiamin and vitamin B12 had lower transcript abundance in the adapted strain (Table S3). Since these two vitamins are provided in the medium used for *S. ovata*, the adapted cells might have reduced the expression of the related biosynthesis pathways genes to limit energy expenses under autotrophic growth conditions. In parallel, a gene coding for the vitamin B12 transporter BtuB had higher transcript abundance in the adapted strain further indicating that the cells were favoring transport of exogenous vitamins over *de novo* biosynthesis.

### Amino acids and osmolytes

Yeast extract is also an important source of amino acids and thus it is not surprising that genes involved in biosynthesis of leucine, valine, and tryptophane had higher transcript abundance in met-T18-2 in the absence of yeast extract (Table S1). This included *nifJ1*, a gene coding for one of the two pyruvate-ferredoxin oxidoreductases (PFOR) of *S. ovata* catalyzing the reversible conversion of pyruvate to acetyl-CoA^48^. During growth on methanol, PFORs of *S. ovata* are likely to convert part of the acetyl-CoA produced by the Wood-Ljungdahl pathway into pyruvate^49^, a precursor molecule in many biosynthetic pathways including amino acids. Other genes participating in the degradation of glutamate, in the deamination of glutamine and asparagine, in the metabolism of glycine, and in the synthesis of pyridoxal 5′ phosphate, a cofactor of multiple enzymes using amino acids as substrates^50^, had lower transcript abundance in the adapted strain (Table S3). Moreover, multiple genes associated with the acetoin metabolism had lower transcript abundance in the adapted strain. This could be related to the role of (S)-2-acetolactate as a precursor molecule in both the acetoin metabolism and the biosynthesis of amino acids. Repressing the expression of acetoin-related genes could increase the flux of (S)-2-acetolactate toward amino acids biosynthesis.

Furthermore, three genes involved in amino acids metabolism or transport were mutated in the adapted strains (Table S1, Fig. 3). Mutations were detected in *ald2*, a gene coding for one of the two alanine dehydrogenases of *S. ovata*, in *grd2*, a gene coding for a subunit of a glycine reductase and in *putP*, a gene coding for a sodium/proline symporter.

Beside protein synthesis, amino acids like proline, glycine along with its methyl derivatives, and glutamate are compatible solutes that can accumulate into the bacterial cells to confer tolerance to osmotic stress, solvents, and thermal stress^51^,^52^,^53^. Mutations and differential expression of genes participating in the metabolism of the osmoprotectants proline and glycine suggest that the adapted *S. ovata* is using them to enhance its tolerance to methanol.

### Cell wall and transporter

**Figure 5:**
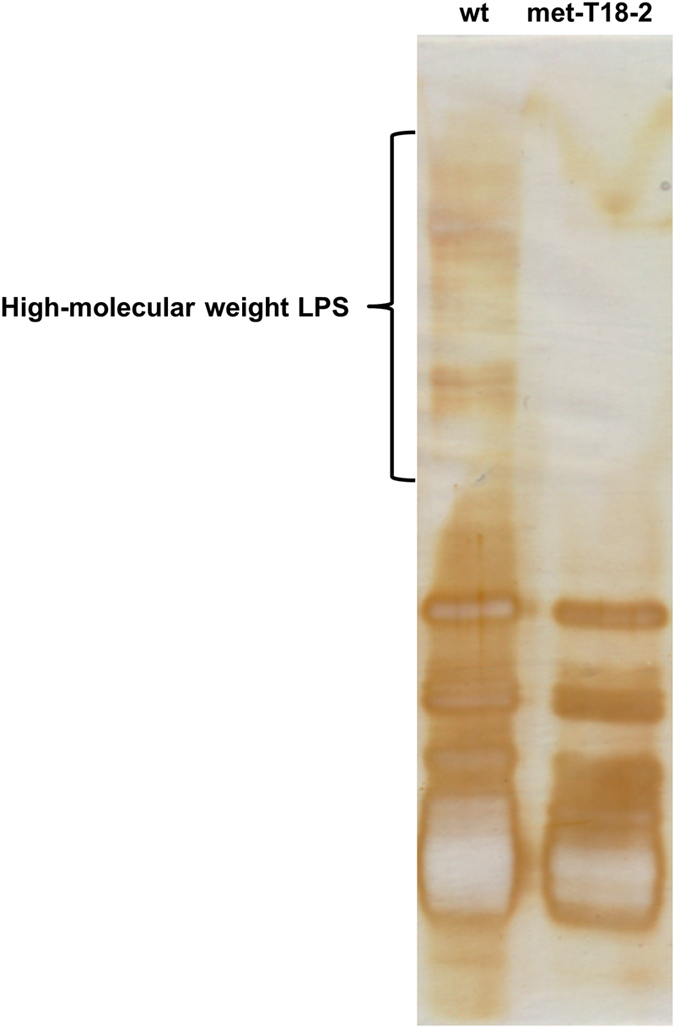
LPS profiles of *S. ovata* wild type (wt) and of the methanol-adapted strain (met-T18-2).

Development of a higher tolerance to solvent in bacteria has been associated with changes in the lipopolysaccharide (LPS) content of the cell envelope^54^,^55^. Lgt2, a gene coding for one of the two prolipoprotein diacylglyceryl transferases of *S. ovata* was mutated in the methanol-adapted strain (Table S1). A SNP causing the substitution of the glycine at position 230 by a glutamic acid appeared at transfer 4. This enzyme attaches a lipid to the preprolipoprotein anchoring it to the cytoplasmic membrane in the first step of the synthesis of mature lipoprotein^56^. Moreover, *rfbG*, a gene participating in the biosynthesis of polysaccharides specific to the O-antigen of LPS, had higher transcript abundance in met-T18-2 (Table S2)^57^. On the contrary, *arnT*, a gene coding for an enzyme modifying LPS by adding a 4-amino-4-deoxy-L-arabinose to the lipid A, had a lower transcript abundance in the adapted strain (Table S3)^58^. Based on these observations, the LPS profiles of the wild type and of met-T18-2 were investigated (Fig. 5). Significantly less high-molecular-weight LPS were detected in the adapted strain. Interestingly, a solvent-tolerant *Pseudomonas putida* strain also exhibits a similar LPS profile^54^ suggesting that the modifications in LPS of *S. ovata* are involved in higher tolerance to methanol.

Changes in membrane transporters and in the overall protein content of the cell wall are also associated with higher tolerance to alcohols^25^,^55^. The genomes of the adapted strains had five mutations in genes coding for proteins involved in transport (Table S1, Fig. 3). Furthermore, multiple genes related to transport function and cell wall maintenance were differentially expressed (Table S2 and S3) highlighting the importance of those cellular processes for the development of an optimal *S. ovata* strain.

### Chaperone proteins

Stress response to solvents involves chaperone proteins performing protein refolding and preventing aggregation^25^,^59^. For example, exposing *Clostridium acetobutylicum* to butanol resulted in the upregulation of genes coding for chaperones including *dnaJ*, *dnaK* and *hsp* genes^60^ Surprisingly, *dnaJ*, *dnaK2*, and *hsp1* had lower transcript abundance in met-T18-2 (Table S3). A recent study by Fan *et al.* suggested that a moderate increase in protein mistranslation and consequently in protein misfolding could improve resistance to oxidative stress probably by activating the general stress response^61^. The lower expression of genes coding for chaperone in the adapted strain might also cause an increase in protein misfolding leading to a better response to methanol-induced stress.

## Conclusions

Results presented here demonstrate that under stress, *S. ovata* can evolve to oxidize methanol faster by modifying its central metabolism with the possible objective of lessening its exposure to this toxic compound. In parallel, the same adapted strain developed a series of molecular mechanisms increasing its tolerance to methanol including a modified cell wall, the possible use of osmoprotectants, and the possible modulation of chaperone activity. This indicates that ALE can be employed to cause the apparition of more than one beneficial characteristic at the same time. With 33 conserved mutations appearing throughout the ALE experiment and a large contingent of differentially expressed genes, it is clear that a complex network of interactions is required for the simultaneous development of several phenotypic traits in *S. ovata* cells.

Methanol is a promising C1 substrate for the development of biotechnologies producing multicarbon commodity chemicals such as amino acids and polyhydroxyalkanoates^27^. One of the positive aspects of acetogens like *S. ovata* compared to other methylotrophs is that they simultaneously reduce CO_2_ from the environment when oxidizing methanol. This can be exploited to develop environment-friendly biotechnologies with high carbon conversion efficiencies using both methanol and the greenhouse gas CO_2_ as substrates.

Furthermore, *S. ovata* is the most efficient microbial catalyst for MES, a promising technology for the production of commodity chemicals from the greenhouse gas CO_2_ and electricity^30^,^31^. Results presented here clearly indicate that adapting *S. ovata* to grow faster autotrophically on methanol produces a strain also significantly more efficient at reducing CO_2_ to organic chemicals in a MES reactor. This illustrates the potential of rational ALE strategies for the optimization of autotrophic microbial catalysts for the electroconversion of CO_2_ into multi-carbon compounds as well as for other industrially-relevant bioprocesses.

## Materials and Methods

### Organism source and growth conditions

*Sporomusa ovata* DSM 2662^62^ was obtained from the Deutsche Sammlung Mikroorganismen und Zellkulturen (DSMZ). *S. ovata* strains were routinely maintained in the DSM 311 medium with 40 mM betaine under a N_2_-CO_2_ (80:20) atmosphere. For all growth conditions presented in this study, casitone, sodium sulfide, yeast extract, and resazurin were omitted from the 311 medium. When *S. ovata* strains were grown with H_2_ as the sole electron donor and CO_2_ as the sole electron acceptor and carbon source, the N_2_-CO_2_ atmosphere was replaced by a H_2_-CO_2_ atmosphere (80:20) at 1.7 atm. For MES experiments, cysteine was also omitted from the 311 medium.

Culture lag time was approximated as the time at which a regression line associated with the exponential growth phase reached the initial OD545 after the addition of the inoculum^63^.

### The ALE experiment

*S. ovata* DSM 2662 was cultivated in two independent ALE experiments in 311 medium with 2% methanol as the substrate. After growth began, a 10% inoculum was transferred to a fresh medium. The following 17 transfers were done when the culture reached the exponential phase. After the 4^th^, 9^th^, and 18^th^ transfer, the cultures were streaked on 311 agar plates to isolate clones. Identity of the clones was verified by PCR.

### Whole-genome sequencing

Genomic DNA was extracted with Easy-DNA gDNA purification Kit (Life Technologies, Carlsbad, CA) from 6 ml of 311 betaine cultures of methanol-adapted *S. ovata* strains met-T4-2, met-T9-1, met-T18-2, and met-T18-3. The genomic libraries were generated with the TruSeq Nano DNA LT Sample Preparation Kit (Illumina Inc., San Diego CA). Briefly, 100 ng of genomic DNA diluted in 52.5 μl TE buffer was fragmented with a Covaris E220 ultrasonicator (Woburn, MA). The ends of fragmented DNA were repaired by T4 DNA polymerase, Klenow DNA polymerase, and T4 polynucleotide kinase. The Klenow exo minus enzyme was then used to add an ‘A’ base to the 3′ end of the DNA fragments. After ligation of the adapters, DNA fragments ranging from 300 to 400 bp were recovered by beads purification. The adapter-modified DNA fragments were enriched by three cycles-PCR. The average size of dsDNA fragments in the libraries was determined with an Agilent 2100 Bioanalyzer. Sequencing was done with a MiSeq Reagent kit v2 (300 cycles) on a MiSeq (Illumina) platform with a paired-end protocol and read lengths of 151 nucleotides. The sequencing reads were then trimmed with Trimmomatic^64^ before being used for variant calling with breseq^65^. The reference genome for the analysis was *Sporomusa ovata* DSM 2662 (NCBI accession ASXP00000000.1). All the samples had an average coverage of at least 30X.

### RNA sequencing

*S. ovata* DSM-2662 wild type and met-T18-2 were grown in triplicate in 100 ml of 311 medium with H_2_ as the electron donor and CO_2_ as the electron acceptor and source of carbon. After reaching the exponential phase, the whole cultures were centrifuged and resuspended in Max Bacterial Enhancement Reagent (Life Technologies). Total RNA was then extracted with TRIzol Max Bacterial RNA Isolation Kit (Life Technologies). Total RNA was further purified with RNeasy Mini Kit (Qiagen, Hilden, Germany) with on-column DNase treatment. A Ribo-Zero rRNA Removal Kit for bacteria (Illumina) was used to reduce the quantity of ribosomal RNAs present in the total RNA sample. The sequencing libraries were prepared in triplicates using a TruSeq RNA Sample Preparation kit (Illumina). Sequencing was done with a MiSeq Reagent kit v2 (300 cycles) on a MiSeq (Illumina) platform with a paired-end protocol and read lengths of 151 nucleotides. Reads mapping, normalization and quantification of transcript abundance was done from the RNA sequencing data with Rockhopper 2.02^66^. The genome of *S. ovata* DSM-2662 was used as the reference for reads mapping. A log_2_ fold change ≥1.15 or ≤−1.15 was used as cutoff. q-values ≤0.015 (Table S2 and S3) or ≤0.02 (Fig. 3) were used as cutoff. The RNA sequencing data have been deposited with the NCBI GEO database under accession number GSE66194.

### CO_2_ reduction to acetate by MES

MES reactors consisting of three-electrode, dual chambered systems were operated at room temperature with *S. ovata* grown in the cathode chamber as described previously^6^,^31^. The graphite stick cathode (36 cm^2^) and anode (36 cm^2^) were suspended in 250 ml of 311 medium in two chambers separated by a Nafion 115 ion-exchange membrane (Ion Power, Inc., New Castle, DE, USA). The cathode was equipped with a potentiostat (ECM8, Gamry Instruments, PA, USA) at –690 mV versus SHE. The cathode chamber was inoculated with 100 ml of hydrogen-grown culture of *S. ovata* wild type or met-T18-2. During the MES experiment, both the cathode and anode chamber were continually bubbled with N_2_-CO_2_ (80:20).

### High-performance liquid chromatography (HPLC)

Methanol and acetate concentrations in *S. ovata* cultures were measured with an HPLC apparatus equipped with a HPX-87H anion exchange column (Bio-Rad Laboratories Inc., California, USA) at a temperature of 30 °C, with 5 mM H_2_SO_4_ as the mobile phase, and a flow rate of 0.6 ml/min. Detection was done with a refractive index detector.

### Acetate kinase assay

*S. ovata* cultures were grown under a H_2_-CO_2_ atmosphere until reaching the exponential phase. Then,the bacterial cells were centrifuged, washed, and concentrated 10 times in 50 mM Tris-HCl pH 7.4 with 1 mM DTT before being sonicated. The acetyl-P to acetate activity of the acetate kinase enzyme was measured in the whole-cell lysates with the method of Bowman *et al.* (1976) linking the formation of ATP to the reduction of NADP^+^ via two reactions catalyzed by the hexokinase and the glucose-6-phosphate dehydrogenase^67^. Briefly, the reaction contained 100 mM Tris-Cl pH 7.4, 5 mM ADP, 10 mM MgCl_2,_ 5.5 mM glucose, 1 mM NADP^+^, 2 mM DTT, 6 U of hexokinase, 3 U of glucose-6-phosphate dehydrogenase, and whole-cell lysate. The reaction was conducted at room temperature and was started by the addition of 10 mM acetyl-P. The extinction coefficient at 340 nm for NADP^+^ is 6.22 mM^−1^ cm^−1^. Acetate kinase activity was measured as nmol of NADP^+^ reduced min^−1^ mg protein^−1^. Protein concentration in whole-cell lysates was measured with the Pierce Coomassie Plus Assay Kit (Life Technologies).

### LPS extraction and SDS-PAGE

LPS from 20 ml of H_2_-CO_2_-grown *S. ovata* cultures at an OD545 of ca. 0.24 were extracted with the LPS extraction kit from iNtRON Biotechnologies (Korea). 5 μl of LPS from *S. ovata* wild type and from *S. ovata* met-T18-2 were loaded on a 12.5% SDS-PAGE. The gel was silver-stained with the SilverXpress Silver Staining Kit from Life Technologies.

## Additional Information

**How to cite this article**: Tremblay, P.-L. *et al.* Adaptation of the autotrophic acetogen *Sporomusa ovata* to methanol accelerates the conversion of CO_2_ to organic products.. *Sci. Rep.* **5**, 16168; doi: 10.1038/srep16168 (2015).

## Supplementary Material

Supplementary Information
