# Supplementary material for: Adaptation of the autotrophic acetogen Sporomusa ovata to methanol accelerates the conversion of CO2 to organic products
Source: Sci Rep. 2015 Nov 4;5:16168. doi: 10.1038/srep16168 (PMC4632017; doi:10.1038/srep16168)
Supplement: Supplementary Information [file srep16168-s1.pdf]

Supplementary information

Title: Adaptation of the autotrophic acetogen *Sporomusa ovata* to methanol accelerates the conversion of CO_2 to organic products.

Author: Pier-Luc Tremblay, Daniel Höglund, Anna Koza, Ida Bonde, Tian Zhang

Table S1. Mutations common to clones isolated from two independent adaptation experiments at transfer 18 on 2% methanol.

Gene	Annotation	Mutation	Function
**Transfer 4[a]**			
dosC	diguanylate cyclase DosC	CDS[b] 1271/1602 nt (→A[c])	Regulation
SOV_1c02880	acetyltransferase, GNAT family	F114S (TTT→TCT)	
lgt2	prolipoprotein diacylglyceryl transferase	G230E (GGA→GAA)	LPS synthesis
SOV_1c10590	phage/plasmid primase, P4 family	F434L (TTC→TCC)	DNA-related
nifB1	FeMo cofactor biosynthesis protein	Synonymous mutation	Nitrogen fixation
ald2	alanine dehydrogenase	S130P (TCC→CCC)	Alanine metabolism
SOV_2c01490	amino acid/polyamine/organocation transporter, APC superfamily	L374P (CTC→CCC)	Transport
SOV_2c03980/ SOV_2c03990	plasmid maintenance system antidote protein/SOS-response transcriptional repressor	Intergenic -14/-130 (C→T)	DNA-related
dppC1	dipeptide transport system permease protein DppC	M226I (ATG→ATA)	Transport
SOV_2c04040	hypothetical protein	CDS 326/393 nt (→A)	
SOV_2c04050	virulence protein	K347STOP (AAA→TAA)	
SOV_2c08180	radical SAM domain-containing protein	CDS 753/1224 nt (→A)	
grdD2	glycine/sarcosine/betaine reductase complex component C subunit alpha	G84E (GGG→GAG)	Glycine metabolism
topB2	DNA topoisomerase 3	CDS 1356/2010 nt (Δ1bp)	DNA-related
SOV_4c05280	TonB-dependent receptor plug	Synonymous mutation	Transport
SOV_5c00350	peptidase MA superfamily	A415T (GCT→ACT)	Proteolysis
stc10	signal-transduction and transcriptional-control protein	CDS 1277/2007 nt (→G)	Regulation
SOV_7c00540	hypothetical protein	Synonymous mutation	
**Transfer 9**			
SOV_1c06310	radical SAM domain containing protein	CDS 450/1362 nt (→T)	
ackA	Acetate kinase	R176Q (CGG→CAG)	Central metabolism
polC1	DNA polymerase III	E587K (GAG→AAG)	DNA-related
SOV_2c01490/ *feoB5*	amino acid/polyamine/organocation transporter, APC superfamily/ferrous iron transport protein B	Intergenic -39/+573 (Δ1bp)	Transport
SOV_2c04150	sigma54 specific transcriptional regulator, Fis family	CDS 1254/1914 nt (→G)	Regulation
SOV_2c05090	PAS domain S-box/diguanylate cyclase (GGDEF) domain-	G97R (GGG→AGG)	Regulation

	containing protein		
SOV_2c05170	type I restriction-modification system methyltransferase subunit	R434Q (CGG→CAG)	DNA-related
glgA	glycogen synthase	G477W (GGG→TGG)	Glycogen metabolism
mtaC (Sov_3c00470)	corrinoid methyltransferase protein	V18A (GTA→GCA)	Central metabolism
whiA	sporulation transcription regulator WhiA	G111E (GGA→GAA)	Regulation
putP	sodium/proline symporter	M57T (ATG→ACG)	Proline transport
SOV_3c06330	methylase involved in ubiquinone/menaquinone biosynthesis	C195R (TGC→CGC)	
SOV_6c00760	hypothetical protein	CDS 45/207 nt (→A)	
**Transfer 18**			
hxuA2	heme/hemopexin-binding protein	CDS 2455/8160 nt (→G)	Heme acquisition
SOV_2c02350	recombinase	CDS 153/1680 nt (→A)	DNA-related

[a]First transfer at which the mutation was detected by whole-genome sequencing.
[b]The mutation was found in the coding sequence (CDS) of the gene.
[c]The indicated nucleotide was inserted.

Table S2. Genes with higher transcript abundance in the *S. ovata* methanol-adapted strain met-T18-2 compared to the wild type.[a,b,c]

**Locus**	**Annotation**	**Expression wt**[d]	**Expression met-T18-2**[e]	**Log_2 fold change**
SOV_7c00530	aspartate carbamoyltransferase-PyrB	17	275	4.02
SOV_7c00520	dihydroorotase-PyrC2	17	261	3.94
SOV_7c00480	Dihydroorotate dehydrogenase-PyrD	19	282	3.89
SOV_7c00460	orotate phosphoribosyltransferase-PyrE	37	539	3.86
SOV_7c00470	orotidine 5'-phosphate decarboxylase-PyrF2	26	365	3.81
SOV_7c00500	carbamoyl-phosphate synthase large chain CarB	26	298	3.52
SOV_7c00490	dihydroorotate dehydrogenase B (NAD(+)), electron transfer subunit PyrK	29	317	3.45
SOV_7c00510	carbamoyl-phosphate synthase pyrimidine-specific small chain PyrAA	29	284	3.29
SOV_2c03010	response regulator of the LytR/AlgR family	9	88	3.29
SOV_7c00120	nodulation protein NolG	8	67	3.08
SOV_2c03020	hypothetical protein	9	71	2.98
SOV_7c00110	polyketide biosynthesis acyltransferase PksD	14	104	2.89
SOV_2c02990	putative peptidase C11, clostripain familiy	35	247	2.82
SOV_7c00190	sensory transduction protein LytT	2	13	2.70
SOV_7c00580	hypothetical protein	13	83	2.67
SOV_4c04870	magnesium-chelatase subunit ChlI	2	12	2.58
SOV_3c08140	2-isopropylmalate synthase 2-LeuA3	16	89	2.47
SOV_7c00030	sensor histidine kinase ResE	16	57	1.83

SOV_7c00650	Zn-dependent hydrolase, including glyoxylase	8	26	1.70
SOV_3c09310	glycine betaine transporter OpuD	1	3	1.58
SOV_1c05750	hypothetical protein	1	3	1.58
SOV_5c03910	glucose/sorbosone dehydrogenase	1	3	1.58
SOV_1c01610	TonB-dependent receptor	1	3	1.58
SOV_3c09330	prolyl-tRNA editing protein ProX	2	6	1.58
SOV_1c01750	MotA/TolQ/ExbB proton channel	2	6	1.58
SOV_2c01350	ketol-acid reductoisomerase-IlvC	321	951	1.57
SOV_6c02370	ATP phosphoribosyltransferase HisG	256	752	1.55
SOV_5c01620	transporter, hydrophobe/amphiphile efflux-1 HAE1 family	64	186	1.54
SOV_1c05980	pyruvate-flavodoxin oxidoreductase-NifJ1	524	1516	1.53
SOV_2c11200	isopropylmalate/citramalate isomerase small subunit-LeuD3	11	30	1.45
SOV_2c10230	30S ribosomal protein S4	381	1031	1.44
SOV_5c01170	vitamin B12 transporter BtuB	3	8	1.41
SOV_1c01760	biopolymer transport protein	3	8	1.41
SOV_2c03000	signal transduction histidine kinase	34	87	1.36
SOV_7c00560	putative nucleotidyltransferase	46	117	1.35
SOV_1c07700	CoB--CoM heterodisulfide reductase iron-sulfur subunit A2-HdrA	1057	2510	1.25
SOV_4c03900	tryptophan synthase alpha chain-TrpA	59	139	1.24
SOV_4c03890	tryptophan synthase beta chain 2-TrpB	57	134	1.23
SOV_1c07680	heterodisulfide reductase, C subunit HdrC	4463	10489	1.23
SOV_1c07670	methyltetrahydrofolate:corrinoid/iron-sulfur protein methyltransferase AcsE	4282	10002	1.22
SOV_1c04530	N5-carboxyaminoimidazole ribonucleotide mutase PurE1	124	287	1.21
SOV_1c07640	Ferredoxin	3515	8124	1.21
SOV_3c03250	CDP-glucose 4,6-dehydratase-RfbG	174	402	1.21
SOV_1c07660	corrinoid/iron-sulfur protein small subunit AcsD	3103	7136	1.20
SOV_1c09790	hemolysin activation/secretion protein-FhaC	11	25	1.18
SOV_1c07690	CoB--CoM heterodisulfide reductase subunit B-HdrB	3871	8745	1.17
SOV_3c00210	hypothetical protein	102	229	1.17

[a]Triplicate cultures for each strain were grown with H_2 as the electron donor and CO_2 as the electron acceptor and carbon source.
[b]cut-off 1: log2 fold change ≥ 1.15.
[c]cut-off 2: q-value ≤ 0.01.
[d]wt: S. ovata DSM-2662 wild type.
[e]met-T18-2: Methanol 2%-adapted *S. ovata* strain.

Table S3. Genes with lower transcript abundance in the *S. ovata* methanol-adapted strain met-T18-2 compared to the wild type.[a,b,c]

**Locus**	**Annotation**	**Expression wt**[d]	**Expression met-T18-2**[e]	**Log_2 fold change**
SOV_4c04090	diacetyl reductase/acetoin dehydrogenase-ButA	1583	55	-4.85
SOV_1c07100	cation diffusion facilitator family transporter	65	4	-4.02
SOV_3c06240	putative permease	129	9	-3.84
SOV_1c07090	hypothetical protein	1824	139	-3.71
SOV_3c08300	hypothetical protein	427	36	-3.57
SOV_1c07080	hypothetical protein TIGR03905	93	9	-3.37
SOV_2c06980	hypothetical protein	10	1	-3.32
SOV_3c09240	tRNA-Sec	137	14	-3.29
SOV_3c08310	hypothetical protein	241	25	-3.27
SOV_4c06030	hypothetical protein	9	1	-3.17
SOV_4c06040	putative esterase of the alpha-beta hydrolase superfamily	9	1	-3.17
SOV_2c03550	hypothetical protein	9	1	-3.17
SOV_1c07070	dinitrogenase iron-molybdenum cofactor biosynthesis protein	99	11	-3,17
SOV_5c02270	FAD-dependent pyridine nucleotide-disulfide oxidoreductase DrsE	1264	143	-3.14
SOV_4c02570	hypothetical protein	93	11	-3.08
SOV_2c06280	hypothetical protein	84	10	-3.07
SOV_1c05030	hypothetical protein	65	8	-3.02
SOV_2c03570	phage protein, HK97 gp10 family	8	1	-3.00
SOV_2c03700	putative phage protein	8	1	-3.00
SOV_7c00360	hypothetical protein	562	72	-2.96
SOV_2c03540	hypothetical protein	7	1	-2.81
SOV_2c03530	hypothetical protein	7	1	-2.81
SOV_1c02130	hypothetical protein	1904	275	-2.79
SOV_2c01410	hypothetical protein	54	8	-2.75
SOV_7c00270	radical SAM domain-containing protein	113	17	-2.73
SOV_1c12870	hypothetical protein	146	22	-2.73
SOV_7c00450	phosphomethylpyrimidine synthase-ThiC2	119	18	-2.72
SOV_7c00400	acetoin catabolism protein X	691	105	-2.72
SOV_5c02280	transcriptional regulator, ArsR family	4302	654	-2.72
SOV_3c09220	glycine/sarcosine/betaine reductase complex component A GrdA2	131	20	-2.71
SOV_7c00260	aldolase-like protein	111	17	-2.71
SOV_5c01120	phospholipid methyltransferase	19	3	-2.66
SOV_2c06270	hypothetical protein	88	14	-2.65
SOV_7c00380	AhpD domain-containing protein	528	84	-2.65
SOV_5c04070	18 kDa heat shock protein-Hsp2	9860	1575	-2.65

SOV_7c00430	hypothetical protein	568	92	-2.63
SOV_7c00410	acetoin:2,6-dichlorophenolindophenol oxidoreductase subunit beta-AcoB	696	114	-2.61
SOV_7c00250	hypothetical protein	61	10	-2.61
SOV_7c00350	lipoyl(octanoyl) transferase LipB	450	74	-2.60
SOV_2c03610	phage major capsid protein, HK97 family	12	2	-2.58
SOV_2c01520	Fe2+ transport system protein A	30	5	-2.58
SOV_1c03610	anaerobic dehydrogenase	6	1	-2.58
SOV_2c06260	hypothetical protein	403	68	-2.57
SOV_7c00370	lipoyl synthase LipA	458	78	-2.55
SOV_2c01510	Fe2+ transport system protein A	41	7	-2.55
SOV_7c00330	glycine cleavage system H protein	197	34	-2.53
SOV_5c04930	AraC family transcriptional regulator	52	9	-2.53
SOV_2c01500	ferrous iron transport protein B	92	16	-2.52
SOV_3c05030	putative DNA-binding protein	1353	239	-2.50
SOV_7c00420	acetoin:2,6-dichlorophenolindophenol oxidoreductase subunit alpha-AcoA	679	122	-2.48
SOV_4c05420	TonB family protein	55	10	-2.46
SOV_1c05020	transposase IS200-family protein	33	6	-2.46
SOV_7c00340	lipoate-protein ligase A LipA1	259	48	-2.43
SOV_1c07030	hypothetical protein	194	36	-2.43
SOV_7c00390	ihydrolipoyllysine-residue acetyltransferase component of acetoin cleaving system AcoC	459	88	-2.38
SOV_6c01810	short-chain type dehydrogenase/reductase VdlC	46	9	-2.35
SOV_5c03820	18 kDa heat shock protein-Hsp1	16529	3256	-2.34
SOV_1c05060	hypothetical protein	76	15	-2,34
SOV_7c00320	glycolate oxidase subunit GlcD	176	35	-2,33
SOV_4c05270	transcriptional regulator	10	2	-2,32
SOV_7c00310	CoB--CoM heterodisulfide reductase 2 iron-sulfur subunit D-hdrD	105	21	-2,32
SOV_2c01590	hypothetical protein	4060	822	-2,30
SOV_3c09210	glycine betaine transporter OpuD	74	15	-2,30
SOV_5c02790	hypothetical protein	294	60	-2,29
SOV_1c01200	chaperone protein DnaK2	1072	219	-2,29
SOV_4c05440	MotA/TolQ/ExbB proton channel	87	18	-2,27
SOV_1c03590	transcriptional regulator	176	37	-2,25
SOV_7c00290	lipoate-protein ligase A LplA	90	19	-2,24
SOV_1c01190	chaperone protein DnaJ	920	195	-2,24
SOV_2c05080	transcriptional regulator XRE family	97	21	-2,21
SOV_5c02780	molybdenum storage protein subunit beta-MosB	377	82	-2,20
SOV_1c05040	hypothetical protein	55	12	-2,20
SOV_2c01600	hypothetical protein	2431	533	-2,19

SOV_1c02740	branched-chain amino acid permease protein/azaleucin resistance-AzlC	95	21	-2,18
SOV_2c03330	hypothetical protein	501	112	-2,16
SOV_3c09200	selenide, water dikinase-SelD	49	11	-2,16
SOV_1c02100	ferredoxin domain containing protein	381	86	-2,15
SOV_1c07050	putative transporter component	256	58	-2,14
SOV_1c03600	hypothetical protein	261	60	-2,12
SOV_5c00790	transcriptional regulator, MarR family	1592	369	-2,11
SOV_2c10870	hypothetical protein	30	7	-2,10
SOV_1c01990	pyridoxine kinase PdxK	77	18	-2,10
SOV_1c05710	alcohol dehydrogenase, iron-type-Adh	247	58	-2,09
SOV_1c00430	hypothetical protein	34	8	-2,09
SOV_1c06510	flavodoxin-FpaA	17	4	-2,09
SOV_3c08290	putative helix-turn-helix domain containing protein	59	14	-2,08
SOV_3c05660	hypothetical protein	29	7	-2,05
SOV_2c00500	hypothetical protein	115	28	-2,04
SOV_1c12880	4Fe-4S ferredoxin iron-sulfur binding domain-containing protein	86	21	-2,03
SOV_2c06290	cyclic di-GMP phosphodiesterase response regulator RpfG5	141	35	-2,01
SOV_3c06230	small redox-active disulfide protein 2	490	122	-2,00
SOV_4c05280	TonB-dependent receptor plug	12	3	-2.00
SOV_4c02670	hypothetical protein	8	2	-2.00
SOV_3c06520	putative membrane-associated protein-DedA	4	1	-2.00
SOV_2c00290	hypothetical protein	4	1	-2.00
SOV_1c02750	branched-chain amino acid transport protein/azaleucin resistance AzlD	102	26	-1,97
SOV_1c05230	transcriptional regulator, GntR family	94	24	-1,97
SOV_3c09190	selenocysteine-specific elongation factor	31	8	-1,95
SOV_2c06940	hypothetical protein	124	32	-1,95
SOV_4c05430	biopolymer transport protein ExbD/TolR	54	14	-1,95
SOV_1c05240	methylaspartate mutase S chain-MamA	88	23	-1,94
SOV_1c05260	methylaspartate mutase E chain-MutE	107	28	-1,93
SOV_5c02190	putative Zn-dependent protease	733	192	-1,931
SOV_3c03770	hypothetical protein	19	5	-1,93
SOV_3c05650	hypothetical protein	38	10	-1,93
SOV_4c04150	tRNA-Leu	212	56	-1,92
SOV_1c02760	C4-dicarboxylate transporter/malic acid transport protein	106	28	-1,92
SOV_2c11720	hydroxylamine reductase-Hcp1	60	16	-1,91
SOV_4c02090	hypothetical protein	912	244	-1,90
SOV_1c05280	hypothetical protein	237	64	-1,89
SOV_1c07060	sulfur transport	181	49	-1,89

SOV_1c05250	protein MutL	59	16	-1,88
SOV_2c06300	hypothetical protein	199	54	-1,88
SOV_1c05050	transposase IS200-family protein	44	12	-1,87
SOV_1c03630	DMSO reductase anchor subunit	11	3	-1,87
SOV_2c10850	toxin secretion/phage lysis holin	18	5	-1,85
SOV_5c00260	transcriptional regulator, TetR family	36	10	-1,85
SOV_3c07260	2-iminoacetate synthase ThiH	210	60	-1,81
SOV_4c05290	transcriptional regulator, MarR family	302	87	-1,80
SOV_1c00290	glutaminase-asparaginase-AnsB1	147	43	-1,77
SOV_2c06990	putative phage-associated protein	17	5	-1,77
SOV_1c07150	cobyrinic acid ac-diamide synthase	258	77	-1,74
SOV_1c01550	methyl-accepting chemotaxis protein McpA	144	43	-1,74
SOV_3c07290	thiamine biosynthesis protein ThiS	120	36	-1,74
SOV_3c05620	pyridoxal biosynthesis lyase PdxS	783	235	-1,74
SOV_3c08260	hypothetical protein	173	52	-1,73
SOV_1c05370	tRNA-Gly	229	70	-1,71
SOV_5c04760	TonB domain-containing protein	78	24	-1,70
SOV_1c07130	ATPase-like, ParA/MinD	474	146	-1,70
SOV_3c07530	cupin 2 barrel domain containing protein	107	33	-1,70
SOV_1c07170	putative DNA-binding protein	278	86	-1,69
SOV_2c06380	putative two-component system response regulator	135	42	-1,68
SOV_1c08580	integral membrane protein TerC	32	10	-1,68
SOV_2c10860	glycosyl hydrolase family 25	32	10	-1,68
SOV_1c04240	undecaprenyl phosphate-alpha-4-amino-4-deoxy-L-arabinose arabinosyl ArnT	134	42	-1,67
SOV_2c05050	hypothetical protein	331	104	-1,67
SOV_3c05630	glutamine amidotransferase subunit PdxT	765	243	-1.65
SOV_5c01750	ABC transporter, ATP-binding protein	85	27	-1.65
SOV_3c05640	hypothetical protein	393	126	-1.64
SOV_3c05750	phytochrome-like protein Cph2	34	11	-1.63
SOV_1c02230	transcriptional regulator MarR family	34	11	-1.63
SOV_3c09150	glycine/sarcosine/betaine reductase complex component A GrdA1	105	34	-1.63
SOV_1c13360	phospholipase/carboxylesterase	37	12	-1.62
SOV_4c04700	hypothetical protein	74	24	-1.62
SOV_2c06500	tRNA-Ser	150	49	-1,61
SOV_1c07040	coenzyme A disulfide reductase	174	57	-1.61
SOV_2c01270	hypothetical protein	363	119	-1.61
SOV_2c06490	tRNA-Ser	414	136	-1.61
SOV_1c07160	cobyrinic acid ac-diamide synthase	283	93	-1.61
SOV_1c02730	transcriptional regulator, TrmB family	301	99	-1.60
SOV_3c07280	thiamine biosynthesis protein ThiF	170	56	-1.60
SOV_3c07270	thiazole synthase-ThiG	127	42	-1.60

SOV_2c03320	hypothetical protein	144	48	-1.58
SOV_1c12830	hypothetical protein	24	8	-1.58
SOV_2c07290	hypothetical protein	3	1	-1.58
SOV_1c10480	hypothetical protein	3	1	-1.58
SOV_4c04680	putative DNA recombinase CisA4	3	1	-1.58
SOV_2c04590	ABC-type cobalamin/Fe3+-siderophores transport system, ATPase componnent	3	1	-1.58
SOV_4c02350	hypothetical protein	3	1	-1.58
SOV_2c00280	hypothetical protein	3	1	-1.58
SOV_4c01850	hypothetical protein	3	1	-1.58
SOV_3c02610	hypothetical protein	3	1	-1.58
SOV_2c00730	tRNA-Sec	6	2	-1.58
SOV_1c01330	thiamine S protein	306	103	-1.57
SOV_1c13350	regulatory protein BlaR1	83	28	-1.57
SOV_1c10860	peptidoglycan endopeptidase LytF	41	14	-1.55
SOV_5c02480	major facilitator superfamily MFS_1	82	28	-1.55
SOV_5c03170	hypothetical protein	339	117	-1.53
SOV_1c06700	transcriptional regulator	150	52	-1.53
SOV_1c09300	transcriptional regulator, AbrB family	1310	460	-1.51
SOV_2c07730	Flp pilus assembly protein, pilin Flp	262	94	-1.48
SOV_2c03280	hypothetical protein	117	42	-1.48
SOV_2c06870	hypothetical protein	152	55	-1.47
SOV_3c09180	thioredoxin reductase	33	12	-1.46
SOV_2c01540	efflux pump periplasmic linker BepF	22	8	-1.46
SOV_2c00410	FeMo cofactor biosynthesis protein NifB	33	12	-1.46
SOV_1c02960	transcriptional regulator, TetR family	236	86	-1.46
SOV_2c05070	zinc-ribbon domain containing protein	98	36	-1.44
SOV_3c07520	NADPH-dependent FMN reductase	563	208	-1.44
SOV_2c03310	hypothetical protein	154	57	-1.43
SOV_2c07150	hypothetical protein	181	67	-1.43
SOV_3c08280	putative transcriptional regulator	121	45	-1.43
SOV_2c05250	tRNA-Gly	258	96	-1.43
SOV_1c00890	transposase IS66	439	164	-1.42
SOV_3c06300	hypothetical protein	56	21	-1.42
SOV_2c03830	hypothetical protein	8	3	-1.42
SOV_2c10720	hypothetical protein	85	32	-1.41
SOV_2c02320	hypothetical protein	77	29	-1.41
SOV_1c04250	transcriptional activator protein IrlR	53	20	-1.41
SOV_1c05070	transposase IS200-family protein	63	24	-1.39
SOV_5c04540	LysM domain containing protein	147	56	-1.39
SOV_1c01370	hypothetical protein	110	42	-1.40
SOV_2c09460	transcriptional regulator CtsR	413	158	-1.39
SOV_1c02090	transcriptional regulator, HxlR family	140	55	-1.35

SOV_1c09530	hypothetical protein	101	40	-1.34
SOV_4c03480	chemotaxis protein CheA	37	15	-1.30

[a]Triplicate cultures for each strain were grown with H_2 as the electron donor and CO_2 as the electron acceptor and carbon source.
[b]cut-off 1: log2 fold change ≤ -1.15.
[c]cut-off 2: q-value ≤ 0.01.
[d]wt: S. ovata DSM-2662 wild type.
[e]met-T18-2: Methanol 2%-adapted *S. ovata* strain.
